# Supplementary material for: The Impact of High-Pressure Processing on Physicochemical Properties and Sensory Characteristics of Three Different Lamb Meat Cuts
Source: Molecules. 2020 Jun 8;25(11):2665. doi: 10.3390/molecules25112665 (PMC7321250; doi:10.3390/molecules25112665)
Supplement: Supplementary file 1 [file molecules-25-02665-s001.pdf]

Supplementary Table 1. Kaiser Meyer Olkin (KMO) sampling adequacy of measure for fatty acid molecules.

Fatty Acids	KMO
C16:1	0.702
C17:1	0.743
18:1n9	0.708
18:2n6	0.777
18:3n3	0.689
18:3n6	0.678
22:2n6	0.564
20:4n6	0.269
20:5n3	0.708
C16:0	0.686
C17:0	0.851
C18:0	0.849
C20:0	0.703
C21:0	0.742
C22:0	0.824
C23:0	0.225
C24:0	0.732
SFA	0.826
MFA	0.729
PUFA	0.792
P:S	0.806
n-3	0.757
n-6	0.740
n-6/n-3	0.468
TBARS	0.409
Overall KMO	0.717

Supplementary Table 2. Kaiser Meyer Olkin (KMO) sampling adequacy of measure for amino acid molecules.

Amino acids	KMO
ASP	0.883
GLU	0.756
PHE	0.861
TRP	0.868
TYR	0.857
HIS	0.460
LYS	0.716
ALA	0.448
GLY	0.802
ILE	0.849
LEU	0.773
PRO	0.704
VAL	0.762
THR	0.722
SER	0.773
MET	0.723
Overall KMO	0.773
